# Folic Acid, Folinic Acid, 5 Methyl TetraHydroFolate Supplementation for Mutations That Affect Epigenesis through the Folate and One-Carbon Cycles

**DOI:** 10.3390/biom12020197

**Published:** 2022-01-24

**Authors:** Yves Menezo, Kay Elder, Arthur Clement, Patrice Clement

**Affiliations:** 1 Laboratoire CLEMENT, Avenue d’Eylau, 75016 Paris, France; aclement@laboclement.com (A.C.); pclement@laboclement.com (P.C.); 2 Bourn Hall Clinic, Cambridge CB232TN, UK; kay.elder@bourn-hall.com

**Keywords:** methylation, folic acid, folinic acid, 5MTHF, UMFA, fertility, cancer, epigenesis

## Abstract

Methylation is an essential biochemical mechanism that is central to the transmission of life, and crucially responsible for regulating gametogenesis and continued embryo development. The methylation of DNA and histones drives cell division and regulation of gene expression through epigenesis and imprinting. Brain development and its maturation also depend on correct lipid methylation, and continued neuronal function depends on biogenic amines that require methylation for their synthesis. All methylation processes are carried out via a methyltransferase enzyme and its unique co-factor S-adenosylmethionine (SAM); the transfer of a methyl group to a target molecule results in the release of SAH (SA homocysteine), and then homocysteine (Hcy). Both of these molecules are toxic, inhibiting methylation in a variety of ways, and Hcy recycling to methionine is imperative; this is achieved via the one carbon cycle, supported by the folates cycle. Folate deficiency causes hyperhomocysteinaemia, with several associated diseases; during early pregnancy, deficiency interferes with closure of the neural tube at the fourth week of gestation, and nutraceutical supplementation has been routinely prescribed to prevent neural tube defects, mainly involving B vitamins, Zn and folates. The two metabolic pathways are subject to single nucleotide polymorphisms that alter their activity/capacity, often severely, impairing specific physiological functions including fertility, brain and cardiac function. The impact of three types of nutraceutical supplements, folic acid (FA), folinic acid (FLA) and 5 Methyl THF (MTHF), will be discussed here, with their positive effects alongside potentially hazardous secondary effects. The issue surrounding FA and its association with UMFA (unmetabolized folic acid) syndrome is now a matter of concern, as UMFA is currently found in the umbilical cord of the fetus, and even in infants’ blood. We will discuss its putative role in influencing the acquisition of epigenetic marks in the germline, acquired during embryogenesis, as well as the role of FA in the management of cancerous disease.

## 1. Introduction

Folates are crucial to life, as a component and catalyst for essential biochemical reactions, particularly and especially via their central role in the metabolism of nucleotides for DNA synthesis and methylation processes [1].

Methylation is a universal biochemical reaction that covalently adds methyl groups to a variety of molecular targets. It plays a critical role in two major global regulatory mechanisms—epigenetic modifications and imprinting—principally via tagging histones and DNA with a methyl group. The process of imprinting relies on methylation to determine non-equivalent and complementary regulatory characteristics of the male and female genomes. In addition, lipid methylation in brain tissue, together with biogenic amines that also require methylation for their synthesis, are essential for neurodevelopment and regulation of the psychic equilibrium. Availability of these essential methyl groups is linked to the folate/one-carbon cycles: folate deficiency has an impact on the stability of DNA [1], with profound downstream consequences.

Folates are intermediary metabolites in the folate cycle (FC), which is linked to and supports the one carbon cycle (1-CC). Together, these two metabolic pathways are responsible for generating methyl groups and regulating all processes involved in methylation, and thus epigenetic modifications and imprinting. The two cycles are also directly or indirectly implicated in numerous other linked metabolic processes that regulate cell division and tissue development; maintaining the appropriate balance of substrates and cofactors is essential for correct homeostasis, as disruption of an enzymatic step in either cycle can have significant adverse consequences. This is particularly important during early pregnancy, where folate deficiency has an impact on neurodevelopment and placental growth. Altered methylation patterns in placental genes have an effect on fetal growth and development.

5-MTHF is the main form of dietary folate, and represents the predominant physiologic form of folate found in blood and in umbilical cord blood; the availability of 5-MTHF contributes to the conversion of methionine to SAM (S-adenosylmethionine), the universal effector for methylation. After the release of a methionyl group, S-adenosylhomocysteine (SAH) and homocysteine accumulation exert feedback inhibition on the MS enzyme, inhibiting methylation. A number of consecutive steps in the two cycles are subject to mutations due to single nucleotide polymorphisms (SNPs) in the methyltetrahydrofolate reductase (MTHFR) enzyme that affect the efficiency of the cycles by decreasing MTHFR activity, compromising methylation reactions via the reduced availability of methionine, and the accumulation of homocysteine.

Folic acid (FA) administered as a dietary supplement is a synthetic compound, and its metabolism requires initial reduction by DHFR in the liver [2]. This enzyme has weak activity, and in conjunction with SNPs that impair the activity of MTHFR, nutritional supplementation with FA can lead to a syndrome now recognized as UMFA: unmetabolized folic acid syndrome. Detectable levels of UMFA occur temporarily in plasma after the consumption of >200 µg FA, with concentrations increasing parallel to that of total FA after supplementation. UMFA has been detected in cord and infant blood, a source of concern due to potential adverse effects on health, as will be further described here [3]. We will outline a rationale for replacing folic acid supplementation (especially at high doses) with 5-MTHF.

## 2. The Folate Family

**Figure 1:**
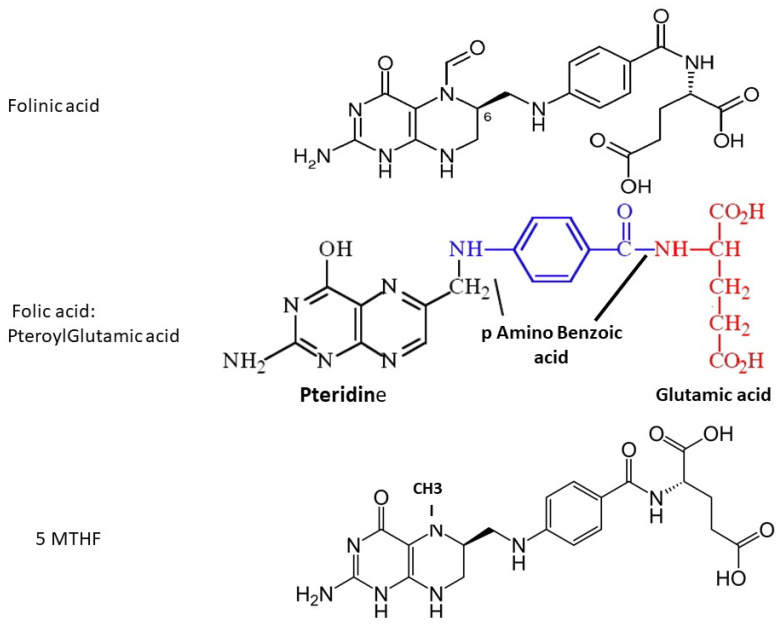
Molecular structure of folinic acid, folic acid and 5-methyltetrahydrofolate; fluorescence-based assays measure the pteroyl group, which is common to all three molecules.

The term ‘folate’ includes several different forms, all of which contain a pteroyl group (see Figure 1). Naturally-occurring folate (Vitamin B9) is a water-soluble molecule that exists physiologically as tetrahydrofolate (THF, the active form) and methyltetrahydrofolate (MTHF, primary form found in blood). Folic acid (FA) is a synthetic manufactured molecule that is used as a dietary supplement and in foodstuff fortification. It is fully oxidized, and is not present in blood unless ingested in food or supplements. Its biological activity depends on the action of dihydrofolate reductase (DHFR) enzyme in the liver, which has unusually slow activity in humans. In order to fulfill a physiological function by entering the folate cycles, FA must first be reduced by DHFR to DiHydroFolate (DHF), and then to tetrahydofolate (THF), before it is converted to the biologically active 5-MTHF.

Folinic acid (Leucovorin) is also a synthetic molecule, a 5-formyl derivative of THF that is readily converted to 5–10 MTHF and 5-MTHF without requiring the action of DHFR. It is used to decrease the toxic effects of chemotherapeutic agents that interfere with folate metabolism by inhibiting DHFR (e.g., methotrexate), and in the co-treatment of other pathologies treated with anti-folate drugs.

### Folate Levels and Folate Assays

Assays that measure folate concentrations in blood are subject to major issues that are often neglected [4]. Fluorescence-based assays measure all “folates” with a pteroyl core, and this includes UMFA, as well as THF, DHF, methyleneTHF, 5-MTHF and FA. When fluorescence assays are used to monitor folate levels after FA is prescribed, “folate” levels are seen to increase, irrespective of their true formula. The results are therefore meaningless, since they do not measure biologically active folates and do not reflect a true physiological status. Assays that are based upon liquid chromatography (+/− mass spectrometry) can measure the three nutraceutical complements that may be prescribed (Figure 1): folinic acid (FLA), folic acid (FA, pteroylglutamic acid) and 5-MTHF, and these should be used in order to determine physiological folate status.

## 3. The One Carbon (1-CC) and the Folates Cycle (FC)

**Figure 2:**
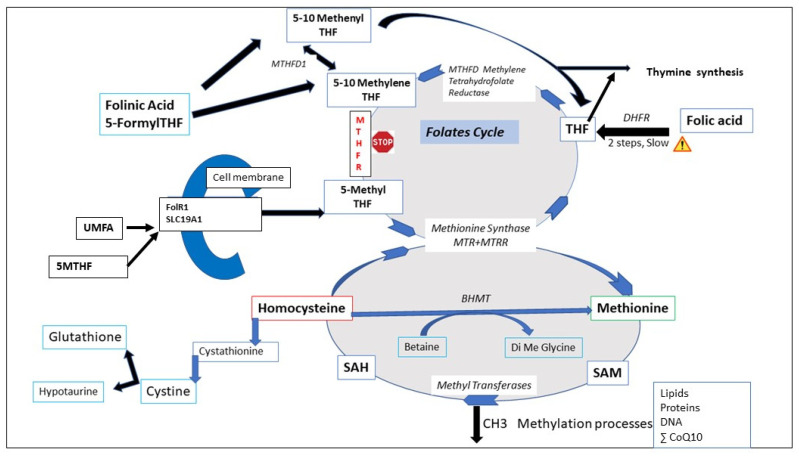
Interactions between the folates cycle and the one-carbon cycle. DHFR: dihydrofalate reductase, MTHFR: methylenetetrahydrofolate reductase. MTHFD1: methylenetetrahydrofolate dehydrogenase 1. MTR: *methionine synthase*, MTRR: *methionine synthase reductase*. SAM: S-adenosylmethionine, SAH: S-adenosylhomocysteine, THF: tetrahydrafolate.

There is a first bottleneck at the entry of the FA into the folates cycle (Figure 2): the DHFR activity needed to form THF is poorly efficient [2]. Then, the second block may occur at the MTHFR level (STOP sign, when the MTHFR SNPs are present, especially 677TT). This leads to an accumulation of 5–10 Methylene THF. Another critical point is the competition between UMFA and 5 MTHF for the receptors and transporters. Mutations at the level of Methionine synthase will impair Hcy recycling. UMFA and 5 MTHF compete to be transported into tissues by the folates transporters (reduced folate carrier and proton-coupled folate transporter) and the folate receptors. THF is an actor in DNA synthesis and DNA repair via the synthesis of purines and thymidylate.

These two cycles are at the epicenter of the methylation process, and also have a role in protecting methyl tags against oxidative stress. Basically, the folates cycle provides the support that allows homocysteine (Hcy) to be recycled to methionine (Met). These two metabolic cycles are subject to hazards imposed by mutations or polymorphisms that impair their metabolic capacity.

Methionine adenosyl transferase (MAT) is the enzyme that catalyzes condensation between adenosine (from ATP) and methionine. S-Adenosyl Hydrolase (AHCY) releases homocysteine from SAH after the methylation processes have been carried out. SAH, one of the proteins that is most highly conserved in living beings, is a potent inhibitor of methylation. Homocysteine is eliminated with the associated formation of cysteine via the cystathionine beta-synthase (CBS) pathway, also known as the cystathionase or transulfuration pathway, cofactor B6; the activity of this pathway is up-regulated by estrogens [5]. Numerous mutations that reduce CBS activity have been described as sources of health issues due to high levels of circulating homocysteine. One of the pathways from the 1-CC transfers methyl groups from betaine to Hcy, catalyzed by the betaine–homocysteine methyltransferase (BHMT) enzyme. This recycling system is generally considered as secondary when compared to the methionine synthase (MS) pathway, but one of the SNPs on the BHMT gene (G716A) is associated with adverse health outcomes. Other mutations on this pathway affect the synthesis of glutathione (gamma glutamyl cysteine ligase, catalytic and regulatory subunits), and this has an effect on redox balance: another link between oxidative stress and methylation anomalies [6].

5-MTHF is the substrate for methionine generation by methionine synthase in the 1-CC (See Figure 2), and reduction in MS activity at this step leads to a number of adverse metabolic consequences as a result of the disruption to the 1-CC.

### 3.1. The Folate Trap

**Figure 3:**
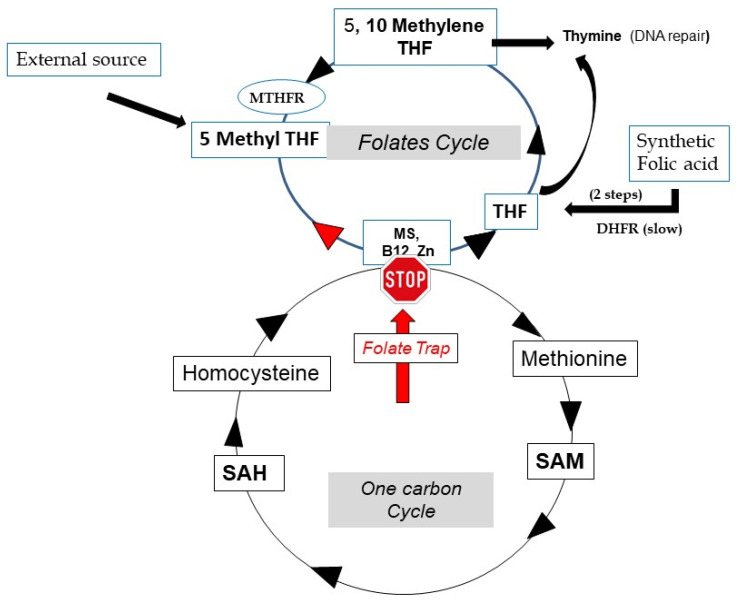
The folate trap: shortage of B12 blocks Methionine synthase activity. Hcy increases, the folate cycle is partly inverted and THF is not re-formed.

The unique biochemical reaction that releases a methyl group from 5-MTHF is catalyzed by the methionine synthase (MS) enzyme: Hcy is the only “methyl acceptor”, allowing methionine to be regenerated as well as THF re-formation, thus perpetuating correct metabolic function of the folate cycle (Figure 3). As B12 is a mandatory cofactor for MS, a deficiency arrests the folate cycle, leading to elevated circulating homocysteine levels, hypermethioninemia, THF shortage and anemia (and infertility). As Zn is also a mandatory cofactor for MS, extreme Zn deficiency can lead to similar pathologies. THF shortage will affect the synthesis of thymidine (from Uridine), also a source of various pathologies including cancers. In this case, MTHF is already present in excess, and folic acid and B12 supplementation are required. The formation of THF, to replete the THF pool, via synthetic FA and DHFR, is slow [2].

### 3.2. 3-MTHFR Variants

As described above, conversion of 5–10 methylene THF to 5-MTHF by MTHFR is a critical step. This enzyme is commonly subject to single nucleotide polymorphisms (SNPs), resulting in multiple variants that effectively reduce its catalytic activity, decreasing the capacity to generate 5-MTFH. Two variants in particular are known to be hazardous: C677T and A1298C, with the latter generally considered to be of lesser risk. A decrease in the activity in terms of 5-MTHF formation can reach −60% for C677T (a thermolabile variant) and −40% for C1298C homozygotes [7,8], causing a reduction in blood folate concentration [9]. Combined heterozygotes C677T/A198C are at risk, and a rather high proportion of the carriers of this combination have an elevated Hcy [10]. These SNPs are highly prevalent as heterozygotes, but homozygous T677T can easily reach 25% in certain populations (Iran, China, Turkey, Spain, Southern Italy). A combination of the two variants as heterozygotes is frequent (>20% in our population: 10), and its presence significantly increases circulating homocysteine levels in men. Combined homozygosity for one variant and heterozygosity for another is rare, but does exist: 0.4% in our population. Combined double heterozygosity was not detected in our population: this combination is probably lethal. However, this combination does not necessarily coincide with elevated circulating homocysteine. Out of >2900 patients tested, we observed that high levels of circulating homocysteine linked to a MTHFR SNP was mostly restricted to males. This sex-related observation is difficult to explain, but may be due to specific up-regulation of the CBS pathway (driven by estrogen) in females. However, this does not solve the problem of inadequate methylation in females, as the formation of methionine from Hcy remains low. Association of the two MTHFR variants with numerous diseases, including cancer, has been demonstrated. In gynecology/obstetrics, the most common severe problem, apart from gamete-related infertility, is an increase in neural tube defects and preterm births, as well as a shift in the morphometric parameters of babies [11]. In very preterm infants, perturbation of DNA methylation elevates prenatal risk factors that are associated with poor health and developmental outcomes [12].

Methionine synthase (Cofactors: Zn + vitamin B12) is a dual protein at the interface between the FC and the 1-CC that combines methionine synthase (MTR) and methionine synthase reductase (MTRR) activities. Several high-risk rare mutations have been described: A2756G is of greatest concern, as it affects global genome methylation [13].

## 4. Metabolism of Synthetic Folic Acid (Pteroylglutamic Acid, FA) and the UMFA Syndrome

In order to enter the folate cycle, FA must first be converted to tetrahydrofolate (THF) via two reducing biochemical steps catalyzed by DHFR—dihydrofolate reductase, (co-factor NADP(H)). This is a rate-limiting step, and DHFR has very weak activity in humans even in the absence of SNP mutations, with considerable inter-individual variation. Therefore, experiments carried out in animal systems (especially rats, where activity can be estimated as 25× higher than in humans), should take this specificity into account. High doses of FA leads to a rapid saturation/inhibition of the DHFR enzyme, leading to an accumulation of un-metabolized folic acid (UMFA) and the UMFA syndrome [2]. These authors confirm that the capacity to metabolize folic acid in humans is low, especially at high FA doses (5 mG or more); the efficacy of prescribing such high doses is questionable, as this may be associated with several pathological issues. Levels of circulating UMFA in the population is persistent in countries where the FA fortification of grains and cereals is implemented [14]. In the BBC (Boston Birth Control) cohort, UMFA was always detected at birth [15]. UMFA may compete with natural folate (MTHF) for the folate transporter (SLC19A1) and the folate receptor (FolR1), thus depleting active folate for participation in the two metabolic cycles. The PCFT-SLC46A1, proton-coupled folate transporter, responsible for transport of folates in the intestine, at low pH; is not detected/active in the early embryos, where all the fluids are at an alkaline bicarbonate-mediated pH. However, this transporter is expressed in fetal tissues as early as ten weeks gestation, at high levels in the intestine of a 20-week old fetus, and in the placenta, and this could be a factor in the appearance of UMFA in cord blood at birth. Tetrahydrofolate (THF) produced by DHFR is converted to 5–10 methylene THF by methylene tetrahydrofolate dehydrogenase (MTHFD1), without specific problems. However, five rare but significant SNPs that affect this step have been described in association with cancers, migraines, congenital anomalies such as neural tube defects, and congenital heart disease [16]. The most hazardous mutation appears to be G1958A. THF/5–10 methylene THF accumulates at the level of MTHFR, and the accumulation of unmetabolized folates upstream from the enzyme may lead to competitive inhibition, leading to Hcy accumulation [17,18]. MTHFR is allosterically inhibited by SAM, and this also means that an excess of 5-MTHF may be deleterious and that circulating Met level is controlled. This level of fine-tuned regulation must be respected: a high consumption of FA may lead to a pseudo- MTHFR deficiency in healthy patients.

## 5. Folinic Acid (5 Formyl THF, FLA)

Folinic acid binds the classical receptor FolR1 and is transported into cells by the solute carrier SLC19A1. Its metabolism leads to the formation of THF and 5, 10 Methylene THF; an important part of these metabolic steps allows the formation of thymidylate, and the failure of this pathway in folate deficiency leads to DNA damage that has been associated with carcinogenesis [1]. Purine nucleotide biosynthesis de novo (PNB) requires two folate-dependent transformylases utilizing formylTHF. FLA is sometimes prescribed as a treatment for problems associated with MTHFR SNPs, but its metabolites do not succeed in compensating for low MTHFR activity. FLA prevents UMFA accumulation by bypassing weak DHFR activity.

## 6. L MethylFolate (5-MTHF)

5-MTHF is the compound produced by MTHFR (co-factors vitamin B2). FA, FLA and 5MTHF share the same transporter and receptor. In vitro experiments have shown that cells with low MTHFR activity require 5-MTHF [19]; 5-MTHF cannot bypass mutations at the levels of methionine synthase or the receptors/transporters.

## 7. Folate Malabsorption

Mutations on the folate R1 receptor are rare, but have been identified as a source of neurovegetative disorders [20].

### 7.1. Pathologies Associated with Folate Metabolism

Dysregulation of folate metabolism has been associated with carcinogenic, hematologic, cardiac and psychiatric pathologies. These do not necessarily feature an increase in circulating Hcy, although this does represent a major additional hazard [21,22]. Organs such as the testis and the ovaries are surrounded by a capsule that provides a partial, controlled barrier to circulating blood, and they experience a transient high demand for folates in a depleted environment. This is especially important in the case of the ovary, when methylation process is exacerbated by the increased level of estrogen during controlled ovarian stimulation [23]. Estrogens are inducers/controllers of methylation resetting [24]. This shortage is exacerbated in the presence of MTHFR SNPs, with an impact on male and female fertility. There is significant sex variation to this feature, and pathologies are not fully linked to increased levels of homocysteine [10]. In recent years, risks associated with FA supplementation and UMFA have also been highlighted.

### 7.2. FA, FLA and Homocysteine

Experiments carried out using treatments with high doses of FA and FLA (15 mG each) [25,26] reported no difference in a 30% decrease in circulating Hcy; however, the genetic status of the patients was not taken into account. Moreover, only 6.5% of the patients had normal baseline Hcy levels. In addition, the recommended dose greatly exceeded the normal “folates” requirement of 500 to 800 µG per day in adults. FLA does not decrease plasma homocysteine levels in newborns who are at risk of ischemic and hemorrhagic stroke due to elevated Hcy [27]. FLA is rarely used in pathologies of reproduction/embryogenesis; however, its positive effect on counteracting the well-known deleterious effects of valproic acid on embryogenesis is controversial.

Three important questions must be addressed: (1) is FA beneficial for the pathological effects of folate deficiency? (2) Can 5-MTHF safely replace FA without side effects? (3) Can FA induce pathological effects, whether or not via the accumulation of UMFA?

### 7.3. FA vs. 5-MTHF

The efficiency and safety of 5-MTHF has been established, including in children [28,29,30,31,32,33,34,35,36,37], and has been demonstrated to be at least as efficient as FA in reducing homocysteine levels in healthy women [38,39]. It is effective in reducing Hcy in men and women of reproductive age who are carriers of the MTHFR T677T variant [31,32,33,34,35,36,37,38]. 5-methylTHF can/could effectively prevent NTDs by improving folate biomarkers in young women during early pregnancy [33]. No large-scale clinical studies have been performed yet. In our program 250+ pregnancies have been initiated, followed by deliveries, with MTHF treatment at a daily dose of 600–800 µG per day [38,39]. This includes women having previously suffered NTDs or miscarriages with FA at 5 mG/Day. 5MTHF supplementation could be a better alternative to FA in reducing the incidence of NTD, especially in countries that do not implement a program of FA fortification. It is at least as effective before and during pregnancy. The wisest options are either to test MTHFR SNPs and prescribe FA in case of no MTHFR SNP, or to prescribe 5MTHF for carriers. Unmetabolized FA in plasma occurs regularly following FA supplementation, but rarely with 5-MTHF [31]. High doses of folic acid can induce a pseudo MTHFR syndrome simply via a Michaelis and Menten effect on enzymes that have a weak/slow activity [18,19,40]. Determining the real impact of UMFA in infants is important: it is a common feature in countries that implement an FA fortification program. Although fortification with folate is clearly important in prevention of NTDs, the question of which molecule is the most appropriate molecule must be considered: 5 MTHF or FA? Current evidence suggests that folinic acid is not the correct choice.

DNA methylation and gametogenesis are intricately linked due to the fact that primordial germ cells are profoundly demethylated and subsequently re-methylated during a later developmental period: prenatal life in males and postnatal development in females. DNA methylation then plays a major role throughout embryo growth; epigenetic alterations will modify both gene and promoter methylation, leading to epipolymorphism. Pathological folate metabolism alters methylation and induces epigenetic instability in the germ line [41]. UMFA has been found in the placenta, cord blood [3,42,43,44] and even in infants, mainly in countries with mandatory FA fortification; this may interfere with the re-methylation of germinal cells. In the mouse, excess FA generates behavioral alterations in offspring and sex-specific changes in methyl metabolism [45]. The fact that alterations are sex-specific suggests a link with time-related differential methylation/epigenetic mark re-setting in males and females.

**Figure 4:**
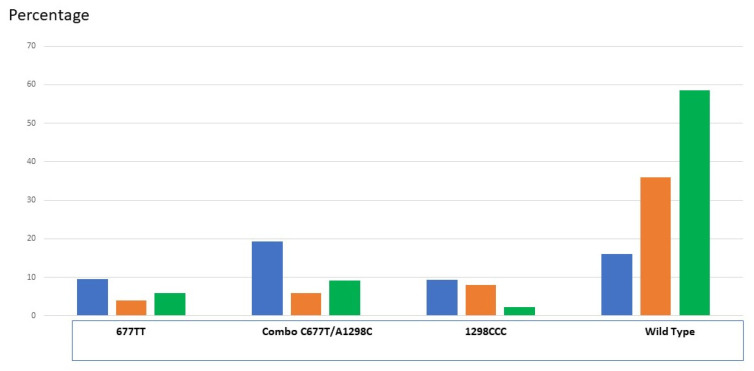
Percentage of the MTHFR C677T and A1298C in our hypo-fertile population (women). Dark blue: hypo-fertile population of various etiologies (1500 women, [10]). Orange: control fertile population (100 patients [49], green, control fertile population (200 patients [48]). Combo: compound heterozygous: C677T/A1298C.

In order to prevent NTDs, nutritional supplementation with 5-MTHF can effectively improve folate biomarkers in young women during early pregnancy [31,34,35]. In adults, C677T SNP is a hazard for spermatogenesis [46], and FA supplementation has been clearly demonstrated as an exacerbating factor in men carrying this SNP [47], for whom the methylome is already compromised. The analysis of SNP distribution shows that T677T is massively over-represented; the incidence of combined heterozygous C677T/A1298C is increased in our sub-fertile couples when compared with two different control groups [48,49]. Symmetrically, there is a higher percentage of wild-type genes in the control fertile population. (Figure 4). Sperm of men carrying MTHFR SNPs have a significant impact on the cytogenetic quality of early embryos [50], with a subsequent increased risk of miscarriage [48,49]. A similar feature can be seen for women [51,52,53]. We have clearly demonstrated that 5-MTHF can provide a solution for problems related to folate cycle metabolism [51,54]; 5-MTHF supplementation can overcome MTHFR SNPs in patients who have failed to conceive even after treatment with high doses of FA, 5 to 15 mg per day (normal adult requirement = 500–800 µg) [51]. Over the past five years, our 5MTHF protocol [51] has succeeded in achieving more than 250 deliveries: pregnancy occurred spontaneously in more than 2/3rds of the 5-MTHF-treated cases, including patients who had suffered several ART failures.

ART (assisted reproduction technology) has highlighted the importance of the methylation process during preimplantation development [55]. In reproductive medicine, 5-MTHF supplementation is preferable to FA. An important point for consideration is the fact that these MTHFR SNPs could induce psychic disorders in the progeny [56,57]: “Early life decides” [57,58]. Moreover, 5 MTHF is an efficient co-treatment in brain pathologies [59], as well as after non-Hodgkin’s lymphoma, probably related to the T677T SNP [60].

Whether or not 5MTHF should/could/must be prescribed to patients carrying the MTHR SNPs during pregnancy—and to their infants/children in order to reduce the risk of psychic or other disorders—remains an open question [61,62].

Endometriosis is an estrogen-dependent inflammatory process that contributes to subfertility, with oxidative stress as a major component of the disease. OS contributes to errors in methylation/epigenetic modifications; this feature is particularly relevant with respect to estradiol-induced epigenetic regulation of gene expression [24]. 5-MTHF supplementation acts as a powerful co-treatment for patients with endometriosis.

### 7.4. Is FA Beneficial for Pathologies Associated with Folate Deficiency?

Observations regarding the impact of FA intake on cardiac and neurologic diseases are confusing; this remains a matter of controversy, and may depend on the doses prescribed [63,64]. FA may have an impact “on mood, arousal, cognitive, and social function [59,65]”. High levels of Hcy are associated with heart disease, and some literature suggests that FA has a positive effects effect; however, the results of supplementation are generally disappointing [63,66,67] and most of the publications neglect the genetic status of the patients. FA supplementation helps to reduce circulating Hcy levels in patients taking antiepileptic drugs [68]. It is also used regularly to combat folate deficiency anemia resulting from the excessive intake of alcoholic beverages, post-bariatric surgery [69], and in conjunction with methotrexate in rheumatoid arthritis [70].

### 7.5. FA, UMFA and Risk of Cancer

The majority of cancers have an epigenetic origin [71], and dysregulation of methylation processes is a relevant issue. MTHFR SNPs increase the risk of cancer; although an association between UMFA and cancer risk is controversial, this may be related to an additional factor created by the fortification of foodstuffs with FA. Breast cancer is one such example: in areas where fortification is not implemented, there is no correlation between UMFA and the incidence of breast cancer, whilst the question of a possible correlation has been raised in areas that do implement fortification [72]. In colorectal cancer, a controversy arises due to the assay methodologies used in calculating doses, as mentioned earlier. Measurements of circulating UMFA using a fluorescence assay does not approach physiological reality, and this is a source of confusion [4,73]. However, FA +B12 administered together may increase the risk of cancer [74]. FA has also been suggested to increase the risk of prostate cancer, but this is controversial [75,76]. However, *in fine*, the FA fortification program could be associated with an additional risk of colon cancer [77]. Overall, it seems that, so far, FA has shown no positive effect in managing cancers, but instead may have a negative impact. At the very least, routine folic acid supplementation should not be recommended as a strategy to combat cancer.

UMFA can be detected in mothers’ milk, and in their infants [3]; since most of the folates share the same receptor and FA competes with 5-MTHF for uptake into the folate cycle [78], this raises an important issue surrounding a potential impact of UMFA on the regulation of epigenetic tags/methylation.

## 8. Conclusions

Methylation is an ineluctable mechanism that regulates a vast number of biochemical/physiological steps throughout life, and the importance of the folate and the one-carbon cycles for correct methylation is often neglected. FA supplementation via nutritional fortification of grain products has been successfully implemented in order to avoid/decrease the incidence of NTDs during pregnancy. However, the generation of circulating UMFA has become a matter of concern, as it may compete severely with the natural “active” folate metabolite, 5-MTHF: this is particularly true for infants. In the assessment of physiological effects via measurement of serum folate levels, fluorescence-based assays measure UMFA as well as other folate molecules, leading to an invalid result and misleading conclusions.

The majority of IVF (in vitro fertilization) units advise patients to take FA supplements prior to their ART cycles, sometimes in high doses (5 to 15 mG): the potential long-term impact of UMFA on germinal cells should be considered. The alteration of epigenetic marks in sperm by UMFA has been observed in animals, and is very likely in humans. Sex-related differential anomalies are clearly linked to the time-related differential resetting of epigenetic marks between males and females [41,61,62,79,80]. The impact on germinal cells is a major concern because the consequences/effects are difficult to perceive [43,79,80,81]: effects on gametogenesis will become evident only in the next generation, after the offspring reproduce, around 25 years later. Moreover, depending on the sex, epigenetic anomalies may skip one generation and reappear later.

Synthetic FA is a true biochemical and physiological challenge for carriers of MTHFR SNPs (especially for infants) as the initial steps of FA metabolism are already jeopardized. 5-MTHF bypasses the narrow initial metabolic step and does not lead to the accumulation of unmetabolized compounds; however, a physiological dose of around 500 µg /day must be respected, and should be increased during the first trimester of pregnancy. In couples presenting with “idiopathic infertility”, the paternal effect must not be overlooked [53,54]: both partners should be routinely tested for MTHFR SNPs, as treatment with 5-MTHFR could be very helpful.

The use of FA supplements in cancer treatments is confusing, but merits attention, as the majority of cancers have an epigenetic origin: the potential impact of UMFA on generating cancer also merits consideration.

In conjunction with the effects of the now permanent and universal presence of endocrine disruptor chemicals (EDCs) in the environment on DNA methylation [82,83,84] covert effects of EDCs combined with the presence of UMFA in body fluids forces a consideration of long-term effects on transgenerational epigenesis, and how this major issue might be remedied.

## Data Availability

Not applicable.
